# The role of different physical function tests for the prediction of fracture risk in older women

**DOI:** 10.1002/jcsm.13508

**Published:** 2024-06-18

**Authors:** Giulia Gregori, Lisa Johansson, Kristian F. Axelsson, Raju Jaiswal, Henrik Litsne, Berit A. M. Larsson, Mattias Lorentzon

**Affiliations:** ^1^ Sahlgrenska Osteoporosis Centre, Department of Internal Medicine and Clinical Nutrition, Institute of Medicine University of Gothenburg Gothenburg Sweden; ^2^ Department of Orthopedics Sahlgrenska University Hospital Mölndal Sweden; ^3^ Region Västra Götaland Närhälsan Norrmalm Health Centre Skövde Sweden; ^4^ Region Västra Götaland Närhälsan Sisjön Health Centre Sisjön Sweden; ^5^ Region Västra Götaland, Sahlgrenska University Hospital Mölndal Sahlgrenska Academy, Sahlgrenska University Hospital Mölndal Sweden

**Keywords:** bone fractures, bone mineral density, epidemiology, osteoporosis, sarcopenia

## Abstract

**Background:**

Physical function is an important risk factor for fracture. Previous studies found that different physical tests (e.g., one‐leg standing [OLS] and timed up and go [TUG]) predict fracture risk. This study aimed to determine which physical function test is the most optimal independent predictor of fracture risk, together with clinical risk factors (CRFs) used in fracture risk assessment (FRAX) and bone mineral density (BMD).

**Methods:**

In total, 2321 women out of the included 3028 older women, aged 77.7 ± 1.6 (mean ± SD), in the Sahlgrenska University Hospital Prospective Evaluation of Risk of Bone Fractures study had complete data on all physical function tests and were included in the analysis. At baseline, hand grip strength, OLS, TUG, walking speed and chair stand tests were performed. All incident fractures were confirmed by X‐ray or review of medical records and subsequently categorized as major osteoporotic fractures (MOFs), hip fractures and any fracture. Multivariate Cox regression (hazard ratios [HRs] and 95% confidence intervals [CIs]) analyses were performed with adjustments for age, body mass index (BMI), FRAX CRFs, femoral neck BMD and all physical function tests as predictors both individually and simultaneously. Receiver operating characteristic (ROC) analyses and Fine and Gray analyses were also performed to investigate associations between physical function and incident fractures.

**Results:**

OLS was the only physical function test to be significantly and independently associated with increased risk of any fracture (HR 1.13 [1.04–1.23]), MOF (HR 1.15 [1.04–1.26]) and hip fracture (HR 1.34 [1.11–1.62]). Adjusting for age, BMI, CRFs and femoral neck BMD did not materially alter these associations. ROC analysis for OLS, together with age, BMI, femoral neck BMD and CRFs, yielded area under the curve values of 0.642, 0.647 and 0.732 for any fracture, MOF and hip fracture, respectively. In analyses considering the competing risk of death, OLS was the only physical function test consistently associated with fracture outcomes (subhazard ratio [SHR] 1.10 [1.01–1.19] for any fracture, SHR 1.11 [1.00–1.22] for MOF and SHR 1.25 [1.03–1.50] for hip fracture). Walking speed was only independently associated with the risk of hip fracture in all Cox regression models and in the Fine and Gray analyses.

**Conclusions:**

Among the five physical function tests, OLS was independently associated with all fracture outcomes, even after considering the competing risk of death, indicating that OLS is the most reliable physical function test for predicting fracture risk in older women.

## Introduction

With an expected increase in the older population in society, the total number of fractures will likely increase.^1^ Fracture care generates high societal costs and is responsible for the second highest hospital expenses after stroke.^2^, ^3^, ^4^ Fractures are also associated with an increased risk of death, especially after hip and vertebral fractures, as well as with other negative consequences such as a worsening quality of life and reduced physical function.^1^, ^5^, ^6^

Multiple risk factors linked to aging, for example, fall risk, reduced physical performance, sarcopenia, bone fragility and general frailty, have been associated with fracture risk.^7^ Bone mineral density (BMD) measured with dual‐energy X‐ray absorptiometry (DXA) is used to diagnose osteoporosis, but BMD alone as a fracture predictor has low sensitivity.^8^ The fracture risk assessment tool FRAX uses several clinical risk factors (CRFs) and BMD as input variables and provides a fracture risk prediction that has higher sensitivity and specificity than BMD alone.^9^, ^10^ Although the FRAX tool is the most validated fracture risk calculator, it is continuously updated to improve and optimize the algorithms with respect to current and novel risk factors.^11^ Recent studies suggest that physical function tests could be incorporated as a novel risk factor for fracture in the FRAX tool.^12^, ^13^

In a study of older men in the Osteoporotic Fractures in Men (MrOS) cohort, it was shown that muscle strength and physical performance measurements, including grip strength, gait speed and chair stand, improve fracture risk prediction beyond the Garvan and FRAX risk calculators, which supports the inclusion of these tests in fracture risk assessment tools.^14^

Among all the physical function tests, hand grip strength has been most extensively studied. In a study of perimenopausal women with normal BMD, grip strength predicted fracture risk.^15^ In the MrOS study, grip strength was found to be associated with increased fall injury risk.^16^

In the Swedish population‐based Sahlgrenska University Hospital Prospective Evaluation of Risk of Bone Fractures (SUPERB) cohort of older women, it was recently demonstrated that both one‐leg standing (OLS) and timed up and go (TUG) predict fracture risk in older women independently of CRFs and BMD,^12^, ^13^ in agreement with other studies that also reported associations between these functional measurements and fracture risk in older adults.^17^, ^18^

Based on the available evidence, it is apparent that physical function tests play an important role in the prediction of fracture risk in both older men and women, although it is still not clear which physical function test is the most reliable predictor.

The aims of the present study were to (i) evaluate the association between physical function tests and the risk of major osteoporotic fracture (MOF), hip fracture and any fracture and (ii) determine which physical function test is the best predictor of fracture risk, together with CRFs used in FRAX and BMD, in older women at high risk of fracture.

## Materials and methods

### Study design and subjects

The SUPERB study is a population‐based prospective study with inclusion performed in Gothenburg, Sweden, between 2013 and 2016.^19^ Overall, 3028 women aged 75–80 years were randomly identified using the National Population Register. Invited participants were contacted by letter and by telephone and asked to participate. Women eligible for inclusion in the study were from the Swedish population and needed to understand Swedish, sign an informed consent form, attend the clinic visit and be ambulatory. The study was approved by the Regional Ethics Review Board in Gothenburg, and the research was performed in accordance with the ethical standards laid down in the 1964 Declaration of Helsinki and its later amendments. The SUPERB study design and procedures are schematically presented in *Figure* *S1*.

### Anthropometrics and dual‐energy X‐ray absorptiometry

Body height and weight were measured using standardized equipment, the same wall‐mounted calibrated stadiometer and scale for all the participants, respectively. The average height and weight were calculated from two consecutive measurements. Assessment of BMD at the femoral neck and lumbar spine (L1–L4) was performed with DXA using Hologic Discovery A devices (Hologic, Waltham, MA, USA). The coefficients of variation (CVs) for women aged 75–80 years old at our facility were 0.7% for lumbar spine BMD, 0.8% for total hip BMD and 1.3% for femoral neck BMD.

### Incident fracture assessment

Incident fractures were verified using X‐ray reports and/or images retrieved from the Regional Digital X‐ray archive responsible for the 49 municipalities in the Region Västra Götaland surrounding Gothenburg, Sweden. A first screening procedure was performed by research nurses, who reviewed all radiology reports between the baseline exam and March 2023. Radiographs without available reports or reports with uncertain fracture diagnoses were further examined by an experienced orthopaedic surgeon (L. J.). Fractures were categorized as any fracture, hip fracture and MOFs, the latter including fractures of the hip, spine, distal forearm and proximal humerus.

### Questionnaires

Each questionnaire was divided into two parts, containing a self‐completed form and a form completed by the research nurse during the visit. CRFs such as current smoking habits,^20^ medical and fracture history, use of medication, accidental falls in the last 12 months, parental history of hip fracture and alcohol consumption obtained using the Alcohol Use Disorders Identification Test (AUDIT) questionnaire^21^ to calculate a variable for excessive consumption (more than 21 standard drinks per week) were covered in the questionnaires. The 12‐Item Short‐Form Health Survey (SF‐12) questionnaire was used to collect information regarding physical activity and self‐reported quality of life, leading to a physical (PCS) and mental (MCS) component score.^22^ Information regarding physical activity habits was collected using the Physical Activity Scale for the Elderly (PASE) survey.^23^

### Physical function tests

Five different physical function tests were conducted at baseline under the supervision of a research nurse or other staff member: hand grip strength, OLS, TUG, walking speed and chair stand test.

1 Hand grip strength

The grip strength was measured for the dominant hand with a Saehan hydraulic hand dynamometer (model SH5001; Saehan Corporation, Masan, Korea). Two measurements were performed with the arm resting on a table and the elbow flexed at 90°. The average of the two measurements (expressed in kilograms) was used in the analyses.^24^

2 One‐leg standing

The clinical balance test (OLS) was performed to measure balance. The participants performed the test with their eyes open, without wearing shoes, with an arm across the chest and standing on one leg with the other leg bent backwards at the knee. After a practice session, the test was performed twice for each leg. The test was stopped if the elevated leg touched the floor, if the arm moved from the chest, if the weight‐bearing limb moved or when the time reached 30 s. The test was performed twice, and the maximum value was used in the analysis.^13^, ^25^

3 Timed up and go

Mobility and balance were tested using the TUG test.^26^, ^27^ The participants started in a sitting position on a chair 45 cm high with armrests. The time was recorded (in seconds) starting when rising from the chair and during the entire procedure: walking 3 m at a normal pace, turning around, walking back and sitting down again. The participants were allowed to use their regular footwear and utilize any mobility aids, if needed.

4 Walking speed

The timed 10‐m walk test^28^, ^29^ was used to measure the gait speed. The participants were instructed to walk 10 m at their own comfortable pace. The time was recorded starting after 2 m and ending at 8 m to eliminate the time required for acceleration and deceleration. The test was performed twice, and the mean value of the two performances was recorded in meters per second.

5 The 30‐s chair stand

The lower body strength was measured using the 30‐s chair stand test.^30^ The participants sat in a chair 45 cm high, with arms crossed against the chest, and on the command ‘go’, they stood up and then returned to a sitting position, repeating this procedure as many times as possible within 30 s.

### Statistical analyses

Baseline characteristics of the study participants were presented with means and standard deviations. Statistical imputation was performed for missing CRF variables (117 [5.0%] women had missing data on one or more CRFs) using the MICE package in RStudio (Multivariate Imputation by Chained Equations), using a single imputation with 10 iterations. In addition to the fracture outcomes, all the other CRFs were included in the imputation. Similar frequencies of CRFs were observed before and after imputation.

To identify which among the five physical function tests (grip strength, OLS, TUG, walking speed and chair stand) could be the best predictor of fracture risk (MOF, hip fracture and any fracture), Cox regression analyses were performed in three steps. First, each physical function test was analysed separately to ascertain its individual association with fracture risk adjusting for age and body mass index (BMI) in Model 1, with added adjustment for CRFs included in FRAX (previous fracture, parental hip fracture, current smoking, rheumatoid arthritis, oral glucocorticoid use, alcohol consumption and secondary osteoporosis) in Model 2 and then with added adjustment for femoral neck BMD in Model 3. Second, the Cox regression analysis was performed similarly, but with all five functional tests in the same model, in order to investigate if any of the five physical function tests were independently associated with fracture risk. In addition, the competing risks analysis by Fine and Gray and receiver operating characteristic (ROC) analyses were performed using the survival and ROCR packages in R Version 4.3.1 (*Figure* *S2A–C*). The ROC analyses were performed using each physical function test at the time together with age, BMI, the CRFs included in FRAX and femoral neck BMD, and in the final model, with all physical function tests included simultaneously. Third, to estimate the importance of each variable included in the multivariable‐adjusted Cox model with all five physical function tests included, Heller's *R* ^2^ was used, and *R* ^2^ values are presented graphically for all the outcomes.^31^ Finally, the study subject characteristics are presented per tertile of OLS time. Differences between groups were investigated using analysis of variance (ANOVA) with the Bonferroni post hoc test for continuous and normally distributed variables, the Kruskal–Wallis H independent test for continuous and not normally distributed variables and the *χ* ^2^ test for dichotomous variables. The Heller analyses were made using RStudio Version 1.4.1106; all other statistical analyses were made using SPSS Statistic Version 28 (IBM Corporation, Armonk, NY, USA). For all the analyses, *P* < 0.05 was considered statistically significant.

## Results

### Baseline characteristics

The characteristics of the 2321 women (mean ± SD age, 77.7 ± 1.6 years) included in this prospective population‐based study are presented in *Table* *1*. The mean (SD) values of each physical function test were 15.25 (5.36) kg for grip strength, 13.90 (9.60) s for OLS, 7.90 (1.79) s for TUG, 1.32 (0.21) m/s for walking speed and 11.52 (3.66) n/30 s for the chair stand test.

**Table 1:** Baseline characteristics of all subjects included in the SUPERB cohort

Characteristics	
*N*	2321
Age, years	77.5 (76.4, 79.1)
Body mass index, kg/m^2^	25.2 (22.8, 27.9)
Clinical risk factors	
Current smoking^a^, *n* (%)	109 (4.7)
Rheumatoid arthritis^b^, *n* (%)	63 (2.7)
Glucocorticoids p.o.^c^, *n* (%)	75 (3.2)
Prior fracture^d^, *n* (%)	831 (35.8)
Parental hip fracture^e^, *n* (%)	410 (17.7)
High alcohol intake^f^, *n* (%)	10 (0.4)
Bone mineral density T‐score and fracture risk	
Femoral neck BMD T‐score	−1.66 ± 0.8
FRAX MOF without BMD (%)	31.1 (23.4, 39.7)
FRAX hip fracture without BMD (%)	16.9 (11.2, 25.0)
FRAX MOF with BMD (%)	19.8 (14.8, 27.7)
FRAX hip fracture with BMD (%)	7.18 (4.19, 12.9)
Physical function tests	
Grip strength, kg	15.2 ± 5.4
One‐leg standing (OLS), s	11.5 (5.4, 21.6)
Timed up and go (TUG), s	7.6 (6.7, 8.7)
Walking speed, m/s	1.3 ± 0.2
Chair stand, *n*/30 s	11.0 (9.5, 14)
Physical activity and quality of life	
Physical Activity Scale for Elderly (PASE)	104.7 (75.7, 141)
Physical health component score (SF‐12)	50.3 (40.4, 55.5)
Mental health component score (SF‐12)	56.6 (50.2, 59.8)

*Note*: Values are presented as numbers (%) for dichotomous variables, as mean ± standard deviation for continuous and normally distributed variables and as median (interquartile range) for continuous and not normally distributed variables. Complete data with no imputed values are shown. FRAX 10‐year probabilities (%) for hip fracture and major osteoporotic fracture (MOF), with or without bone mineral density (BMD) of the femoral neck, are shown. Abbreviations: SF‐12, 12‐Item Short‐Form Health Survey; SUPERB, Sahlgrenska University Hospital Prospective Evaluation of Risk of Bone Fractures.

^a^ *n* = 2319.

^b^ *n* =2316.

^c^ *n* = 2319.

^d^ *n* = 2316.

^e^ *n* = 2294.

^f^ *n* = 2320.

### Association between physical function tests and fracture risk in older women

The incident fractures were stratified into three groups, that is, MOF, hip fracture and any fracture. During a median (interquartile range [IQR]) follow‐up time of 8.1 (7.3, 8.9) years, there were 579 (24.9%) MOF, 163 (7%) hip and 779 (33.6%) any fractures.

When analysing each of the physical function tests (grip strength, OLS, TUG, walking speed and chair stand) one at a time, Cox proportional hazard models revealed significant associations with all investigated fracture outcomes when adjusting for both Models 1 and 2 (*Table* *2*). With the exception of grip strength, these associations were still significant after adding adjustments for femoral neck BMD (Model 3).

**Table 2:** Association between physical function tests and fracture risk in older women—each test analysed separately

	Grip strength	One‐leg standing	Timed up and go	Walking speed	Chair stand test
	HR (95% CI)	HR (95% CI)	HR (95% CI)	HR (95% CI)	HR (95% CI)
Major osteoporotic fracture					
Model 1	**1.11 (1.01–1.21)**	**1.23 (1.12–1.34)**	**1.46 (1.28–1.67)**	**1.32 (1.20–1.47)**	**1.27 (1.15–1.40)**
Model 2	**1.11 (1.01–1.21)**	**1.22 (1.11–1.34)**	**1.43 (1.25–1.63)**	**1.32 (1.19–1.46)**	**1.25 (1.13–1.38)**
Model 3	1.08 (0.99–1.18)	**1.19 (1.09–1.31)**	**1.35 (1.18–1.54)**	**1.27 (1.15–1.41)**	**1.25 (1.13–1.38)**
Fine and Gray	1.07 (0.98–1.17)	**1.17 (1.06–1.28)**	**1.15 (1.07–1.24)**	**1.18 (1.08–1.28)**	**1.18 (1.09–1.28)**
Hip fracture					
Model 1	**1.19 (1.00–1.41)**	**1.50 (1.25–1.80)**	**1.64 (1.31–2.06)**	**1.65 (1.37–1.98)**	**1.46 (1.21–1.75)**
Model 2	**1.20 (1.02–1.42)**	**1.47 (1.22–1.76)**	**1.60 (1.27–2.02)**	**1.62 (1.34–1.96)**	**1.41 (1.17–1.69)**
Model 3	1.13 (0.95–1.34)	**1.41 (1.17–1.69)**	**1.42 (1.13–1.79)**	**1.49 (1.24–1.79)**	**1.41 (1.17–1.69)**
Fine and Gray	1.10 (0.94–1.30)	**1.35 (1.12–1.61)**	**1.17 (1.02–1.33)**	**1.30 (1.12–1.52)**	**1.29 (1.10–1.50)**
Any fracture					
Model 1	**1.10 (1.02–1.19)**	**1.20 (1.11–1.30)**	**1.42 (1.27–1.60)**	**1.28 (1.17–1.40)**	**1.25 (1.15–1.36)**
Model 2	**1.09 (1.01–1.18)**	**1.19 (1.10–1.29)**	**1.38 (1.22–1.55)**	**1.27 (1.16–1.39)**	**1.22 (1.12–1.33)**
Model 3	1.07 (0.99–1.15)	**1.17 (1.08–1.27)**	**1.31 (1.17–1.47)**	**1.23 (1.13–1.35)**	**1.23 (1.13–1.14)**
Fine and Gray	1.06 (0.98–1.14)	**1.15 (1.06–1.24)**	**1.13 (1.06–1.21)**	**1.15 (1.06–1.23)**	**1.16 (1.08–1.24)**

*Note*: Associations were studied using Cox proportional hazard models. Hazard ratios (HRs) per SD are shown with 95% confidence intervals (CIs). Significant HRs are shown in bold. All physical function test variables were included separately. HR per decreased SD for all physical function tests except timed up and go, for which HR per SD increase is shown. Model 1: Adjusted for age and body mass index. Model 2: Model 1 + clinical risk factors (CRFs) included in FRAX (previous fracture, family history of hip fracture, current smoking, rheumatoid arthritis, oral glucocorticoid use, alcohol consumption and secondary osteoporosis). Model 3: Model 2 + femoral neck bone mineral density. Competing risk analysis by Fine and Gray. *N* = 2321; imputed variables for missing CRFs are used.

### Independent association between physical function tests and fracture risk

All five physical function tests were subsequently included together in the models to identify the most reliable independent predictor of fracture risk (*Table* *3*). Cox proportional hazard models revealed that OLS was the only physical function test to be significantly and consistently associated with increased risk of all fracture outcomes (MOF, hip fracture and any fracture) in all adjusted models (*Table* *3*). In the context of these findings, other factors of interest displayed varying patterns across different tertiles of OLS. These factors included age, BMI, grip strength, chair stand, walking speed, PASE and TUG, which are further described in *Table* *4*. Lower age, lower BMI, higher grip strength, greater number of chair stands per 30 s, faster walking speed, greater PASE and slower TUG were observed with increasing tertiles of OLS. There was no significant difference between tertiles regarding femoral neck BMD T‐score, glucocorticoid use, prevalence of fracture, parental hip fracture and rheumatoid arthritis (*Table* *4*).

**Table 3:** Association between physical function tests and fracture risk in older women—all five tests included in the same analysis

	Grip strength	One‐leg standing	Timed up and go	Walking speed	Chair stand test
	HR (95% CI)	HR (95% CI)	HR (95% CI)	HR (95% CI)	HR (95% CI)
Major osteoporotic fracture					
Model 1	1.01 (0.92–1.11)	**1.15 (1.04–1.26)**	1.14 (0.94–1.39)	1.14 (0.99–1.31)	1.12 (0.99–1.26)
Model 2	1.02 (0.93–1.11)	**1.15 (1.04–1.26)**	1.12 (0.92–1.36)	1.15 (1.00–1.32)	1.10 (0.98–1.24)
Model 3	1.00 (0.91–1.09)	**1.13 (1.02–1.24)**	1.06 (0.87–1.29)	1.14 (0.98–1.31)	**1.14 (1.01–1.28)**
Fine and Gray	1.00 (0.91–1.09)	**1.11 (1.00–1.22)**	1.02 (0.91–1.14)	1.08 (0.96–1.21)	**1.10 (1.00–1.22)**
Hip fracture					
Model 1	1.01 (0.85–1.21)	**1.34 (1.11–1.62)**	0.96 (0.67–1.38)	**1.43 (1.11–1.85)**	1.20 (0.96–1.49)
Model 2	1.04 (0.87–1.24)	**1.33 (1.10–1.60)**	0.95 (0.66–1.37)	**1.43 (1.10–1.84)**	1.17 (0.94–1.45)
Model 3	0.99 (0.83–1.18)	**1.29 (1.06–1.55)**	0.87 (0.61–1.25)	**1.37 (1.06–1.77)**	1.23 (0.99–1.53)
Fine and Gray	0.99 (0.83–1.17)	**1.25 (1.03–1.50)**	0.91 (0.74–1.12)	**1.24 (1.00–1.53)**	1.18 (0.98–1.42)
Any fracture					
Model 1	1.01 (0.94–1.10)	**1.13 (1.04–1.23)**	1.14 (0.96–1.36)	1.11 (0.99–1.25)	1.11 (1.00–1.23)
Model 2	1.01 (0.94–1.10)	**1.13 (1.04–1.23)**	1.10 (0.93–1.32)	1.12 (1.00–1.27)	1.09 (0.99–1.21)
Model 3	0.99 (0.92–1.08)	**1.12 (1.03–1.21)**	1.06 (0.89–1.26)	1.11 (0.98–1.25)	**1.12 (1.02–1.25)**
Fine and Gray	1.00 (0.92–1.08)	**1.10 (1.01–1.19)**	1.02 (0.93–1.13)	1.06 (0.96–1.17)	**1** **.10 (1.01‐1.19)**

*Note*: Associations were studied using Cox proportional hazard models. Hazard ratios (HRs) per SD are shown with 95% confidence intervals (CIs). Significant HRs are shown in bold. All physical function test variables were included simultaneously. HR per decreased SD for all physical function tests except timed up and go, for which HR per SD increase is shown. Model 1: adjusted for age and body mass index. Model 2: Model 1 + clinical risk factors (CRFs) included in FRAX (previous fracture, family history of hip fracture, current smoking, rheumatoid arthritis, oral glucocorticoid use, alcohol consumption and secondary osteoporosis). Model 3: Model 2 + femoral neck bone mineral density. *N* = 2321; imputed variables for missing CRFs are used.

**Table 4:** Baseline characteristics according to tertiles of one‐leg standing (OLS)

Tertile of OLS	1	2	3	*P*
Number of subjects	774	774	773	
OLS, s	4.1 (2.7, 5.4)^a^ ^,^ ^b^	11.4 (8.9, 11.4)^a^ ^,^ ^c^	26.7 (21.6, 30.0)^b^ ^,^ ^c^	<0.001
Age, years	78.2 (76.9, 79.6)^a^ ^,^ ^b^	77.6 (76.5, 79.2)^a^ ^,^ ^c^	77.0 (75.9, 78.3)^b^ ^,^ ^c^	<0.001
Body mass index, kg/m^2^	26.1 (23.7, 29.2)^a^ ^,^ ^b^	25.6 (23.1, 28.5)^a^ ^,^ ^c^	24.1 (21.9, 26.4)^b^ ^,^ ^c^	<0.001
Clinical risk factors				
Current smoking, *n* (%)	48 (6.2)	29 (3.7)	32 (4.1)	0.05
Rheumatoid arthritis, *n* (%)	26 (3.4)	19 (2.5)	18 (2.3)	0.4
Glucocorticoids p.o., *n* (%)	25 (3.2)	23 (3.0)	27 (3.5)	0.85
Prior fracture, *n* (%)	278 (35.9)	278 (35.9)	278 (36)	1.00
Secondary osteoporosis, *n* (%)	212 (27.4)	187 (24.2)	146 (18.9)	<0.001
Parental hip fracture, *n* (%)	144 (18.6)	130 (16.8)	144 (18.6)	0.56
High alcohol intake, *n* (%)	7 (0.9)	0 (0.0)	3 (0.1)	0.02
BMD T‐score and fracture risk				
Femoral neck BMD T‐score	−1.65 ± 0.8	−1.58 ± 0.9	−1.67 ± 0.8	0.09
FRAX MOF without BMD (%)	31.6 (23.6, 40.9)	30.6 (22.9, 38.6)	31.2 (23.3, 39.4)	0.17
FRAX hip fracture without BMD (%)	16.0 (11.1, 26.7)	15.6 (10.9, 23.7)	16.2 (11.6, 25.1)	0.10
FRAX MOF with BMD (%)	21.3 (14.9, 28.7)	19.3 (14.5, 27.1)	19.1 (14.7, 27.1)	0.08
FRAX hip fracture with BMD (%)	8.0 (4.3, 14.2)	6.8 (4.0, 12.3)	6.9 (4.2, 12.5)	0.30
Physical function tests				
Grip strength, kg	14.1 ± 5.3^a^ ^,^ ^b^	15.3 ± 5.2^a^ ^,^ ^c^	16.4 ± 5.3^b^ ^,^ ^c^	<0.001
Timed up and go (TUG), s	8.1 (7.2, 9.4)^a^ ^,^ ^b^	7.6 (6.8, 8.6)^a^ ^,^ ^c^	7.0 (6.3, 8.0)^b^ ^,^ ^c^	<0.001
Walking speed, m/s	1.2 ± 0.2^a^ ^,^ ^b^	1.3 ± 0.2^a^ ^,^ ^c^	1.4 ± 0.2^b^ ^,^ ^c^	<0.001
Chair stand, *n*/30 s	11.0 (9.0, 12.0)^a^ ^,^ ^b^	12.0 (10.0, 13.0)^a^ ^,^ ^c^	12.0 (10.0, 15.0)^b^ ^,^ ^c^	<0.001
PASE	90.5 (62.8, 131.7)^a^ ^,^ ^b^	109 (75.7, 141.0)^a^ ^,^ ^c^	113.2 (83.0, 155.2)^b^ ^,^ ^c^	<0.001

*Note*: Values are presented as numbers (%) for dichotomous variables, as mean ± standard deviation for continuous and normally distributed variables and as median (interquartile range) for continuous and not normally distributed variables. Analysis of variance with the Bonferroni post hoc test was used to test differences between continuous and normally distributed variables, the Kruskal–Wallis H independent test for continuous and not normally distributed variables and the *χ* ^2^ test for dichotomous variables. *N* = 2321; imputed variables for missing clinical risk factors are used. Abbreviations: BMD, bone mineral density; MOF, major osteoporotic fracture; PASE, Physical Activity Scale for the Elderly.

^a^ Significant difference between 1 and 2.

^b^ Significant difference between 1 and 3.

^c^ Significant difference between 2 and 3.

**Figure 1:**
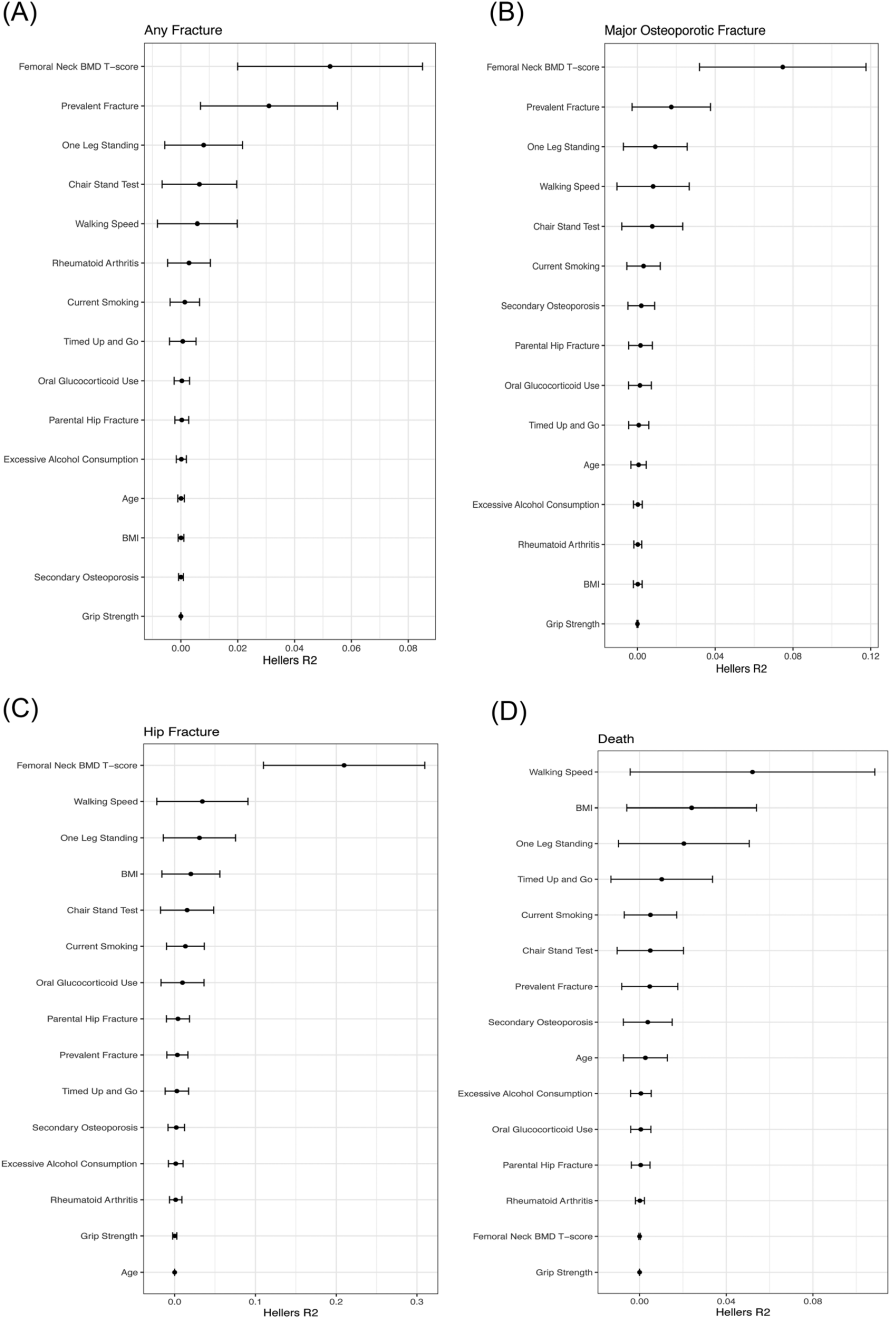
(A–D) Variable importance of risk factors for fractures and death. Heller's *R* ^2^ analysis to estimate the importance of each variable included in the multivariable‐adjusted Cox model. *R* ^2^ values for all covariates in the model are presented graphically. The sum of all the attributable factors was 0.109 (10.9%), 0.127 (12.7%), 0.348 (34.8%) and 0.129 (12.9%) for any fracture, major osteoporotic fracture, hip fracture and death, respectively. BMD, bone mineral density; BMI, body mass index.

In the fully adjusted Cox model (Model 3) with all five physical function tests included simultaneously, using Heller's *R* ^2^, the estimated risk explained (*R* ^2^) by OLS was 0.8% for any fracture and 0.9% for MOF. The corresponding number for hip fracture was 3.1% for OLS and 3.4% for walking speed (*Figure* *1*). Overall, femoral neck BMD was the most important variable, in terms of estimated explained risk, for all fracture outcomes, whilst walking speed was the most important variable for predicting death (*Figure* *1*).

Fine and Gray analysis was also performed to investigate the association between the physical function tests and fracture outcomes when considering the competing risk of death. Although the Fine and Gray‐derived subhazard ratios (SHRs) were in general lower than the corresponding hazard ratios (HRs), OLS, TUG, walking speed and the chair stand test were all significantly associated with any fracture, MOF and hip fracture when considering the competing risk of death (*Table* *2*). In the model including all physical function tests, OLS remained significantly associated with all fracture outcomes when considering mortality (*Table* *3*). A significant association between the chair stand tests was found only for any fracture and MOF, using the Fine and Gray models. After considering the competing risk of death, walking speed was only associated with the risk of hip fracture but not with any fracture or MOF (*Table* *3*).

The associations between baseline physical function tests and incident fractures were also tested using ROC analyses (*Figure* *S2A–C*). The highest area under the curve (AUC) value for any fracture (*Figure* *S2A*) was observed for OLS (0.642), but highly similar AUC values were observed for the chair stand test, TUG, walking speed and grip strength (AUC 0.641, 0.640, 0.640 and 0.635, respectively). For MOF (*Figure* *S2B*), the chair stand test had the highest AUC (0.649), followed by OLS (0.647), TUG and walking speed (0.646) and grip strength (0.639). For hip fracture (*Figure* *S2C*), OLS had the highest AUC (0.732), followed by walking speed (0.730), chair stand test (0.729), TUG (0.724) and grip strength (0.717). Combining all physical function tests resulted in AUC values of 0.652 for any fracture, 0.657 for MOF and 0.743 for hip fracture (*Figure* *S2A–C*).

## Discussion

In this population‐based prospective cohort of older women, we demonstrated that among five tested and commonly used physical function tests, the OLS test was the only test consistently and significantly associated with the risk of MOF, hip fracture and any fracture independently of FRAX CRFs and BMD. In addition, we also found that walking speed predicted incident hip fracture risk independently of covariates and in all used models. These findings indicate that when selecting a physical function test for the clinical evaluation of fracture risk in older women, OLS and walking speed are the most robust tests to use.

Sarcopenia has been defined as a complex medical condition that involves the loss of muscle mass, quality and function of skeletal muscles, a process associated with aging, but with several factors influencing its origin. These factors include lifestyle, genetic, biological (hormonal changes and inflammation) and clinical (diseases).^32^ As the aetiology is multifactorial and the resulting outcomes are often severe and include increased mortality, disability and reduced quality of life, sarcopenia can be defined as a geriatric syndrome, which warrants specific interventions to prevent its associated negative outcomes.^33^, ^34^ In this study, we have tested the association between different physical function tests, which to some degree reflect the geriatric syndrome, and fracture outcomes to identify a risk that might be reversible.

We previously demonstrated in the SUPERB cohort that OLS and TUG predict fracture risk independently of CRFs and BMD.^12^, ^13^ However, these functional parameters were tested one at a time, which does not allow conclusions about which of these tests is the most robust for fracture prediction in the cohort. In this study, we aimed to determine which of all available physical function tests (hand grip strength, OLS, TUG, walking speed and chair stand) was the best performing test in predicting several fracture outcomes. OLS was most robustly associated with all fracture outcomes, regardless of adjustment, but the interdependence between physical function tests was evident, as illustrated by the clear association between OLS, by tertiles, and the other physical function tests as shown in *Table* *4*. Thus, our results indicate that several physical function tests may be important for fracture risk, but we suggest that if choosing a single test, OLS may be the most efficient in capturing the risk of fracture in this population.

Several other studies have presented similar findings regarding the association between OLS and fracture risk, although these have not performed similar extensive adjustments for confounders. Another Swedish study observed that with 1 s longer OLS time, the risk of hip fracture decreased significantly by 5%.^35^ An analysis of a South Korean population of men and women aged 40 years or older demonstrated that OLS predicted the risk of fracture in adults independent of age, hip BMD, fall history and lifestyle factors.^36^ However, neither of these studies tested the performance of multiple physical function tests on fracture risk.

This study has several strengths. This is the first study, to our knowledge, to test most of the commonly used physical function tests simultaneously to identify the most robust predictor of fracture risk in older women. The current study also includes a relatively large, homogeneous population‐based cohort. In addition, X‐rays and radiology reports were used to identify all the fractures, thereby providing highly accurate fracture information. However, this study is limited to only Swedish ambulatory women in a rather narrow age span (75–80 years), indicating that it could be difficult to extrapolate these findings to other populations, for example, men, different age groups and populations with different ethnic backgrounds. Further studies are therefore warranted to determine if OLS can be an effective addition to fracture risk assessment tools, such as the FRAX algorithm, to improve fracture prediction across multiple populations with different characteristics.

In consideration of these findings, OLS could contribute to improving fracture risk assessments at a low cost to the healthcare system, for example, by integrating it at annual primary check‐ups in order to enhance fracture prediction in older women.

In conclusion, results from this study demonstrate that OLS was the only physical function test that was independently and consistently associated with all fracture outcomes, including MOF, hip fracture and any fracture in older women, suggesting that OLS could be a useful physical function test when evaluating fracture risk in older women. However, other tests not included in this study may be equal to or superior to OLS, which was found most compelling in this analysis.

## Conflict of interest statement

No author has a conflict of interest in regard to the content of this manuscript.

## Supporting information


**Figure S1.** SUPERB study design and procedures.
**Figures S2.** (A–C) Receiver operating characteristics (ROC) curves for the physical function tests for the discrimination of any fracture (2A), major osteoporotic fracture (2B), and hip fracture (2C). Additional covariates in the tested models included age, BMI, and clinical risk factors (previous fracture, parental hip fracture, current smoking, rheumatoid arthritis, oral glucocorticoid use, alcohol consumption, secondary osteoporosis, and femoral neck BMD).
