# Supplementary material for: The role of different physical function tests for the prediction of fracture risk in older women
Source: J Cachexia Sarcopenia Muscle. 2024 Jun 18;15(4):1511–9. doi: 10.1002/jcsm.13508 (PMC11294044; doi:10.1002/jcsm.13508)
Supplement: Supplementary file 1 — Figure S1. SUPERB study design and procedures. Figures S2. (A–C) Receiver operating characteristics (ROC) curves for the physical function tests for the discrimination of any fracture (2A), major osteoporotic fracture (2B), and hip fracture (2C). Additional covariates in the tested models included age, BMI, and clinical risk factors (previous fracture, parental hip fracture, current smoking, rheumatoid arthritis, oral glucocorticoid use, alcohol consumption, secondary osteoporosis, and femoral neck BMD). [file JCSM-15-1511-s001.pdf]

Supplemental Figure 1. SUPERB study design and procedures

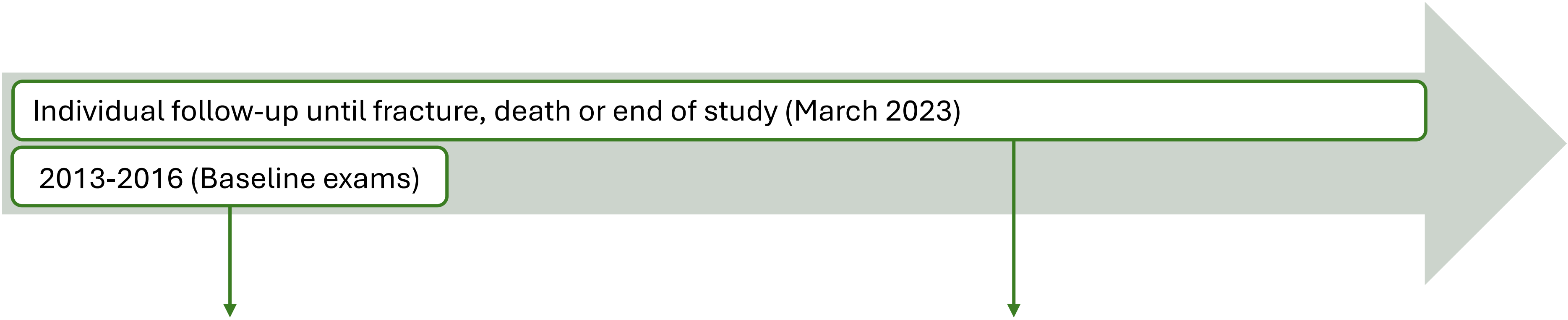

- Physical function tests:
  - One leg standing (OLS),
  - Grip strength,
  - Timed up and go (TUG),
  - Walking speed,
  - Chair stand test
- Questionnaires
- Anthropometrics
  - Height, weight, body mass index (BMI)
- Dual x-ray absorptiometry (DXA) scans
  - Appendicular lean mass

- Deaths retrieved from the regional population registry Västfolket
- X-ray verified fractures retrieved from the regional digital X-ray archive

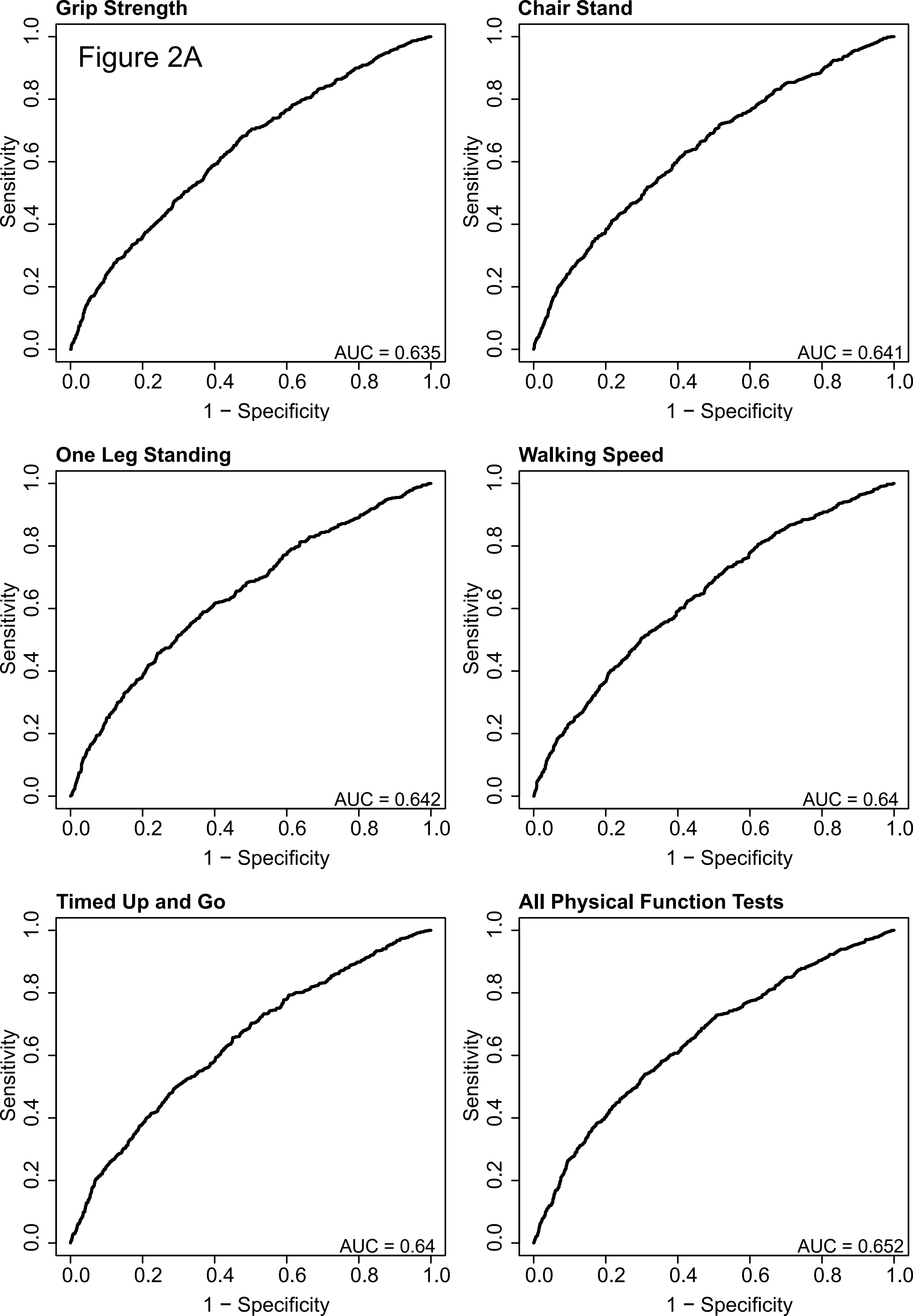

Figure 2A
Grip Strength
AUC = 0.635
Chair Stand
AUC = 0.641
One Leg Standing
AUC = 0.642
Walking Speed
AUC = 0.64
Timed Up and Go
AUC = 0.64
All Physical Function Tests
AUC = 0.652
Sensitivity
1 − Specificity
0.0
0.2
0.4
0.6
0.8
1.0

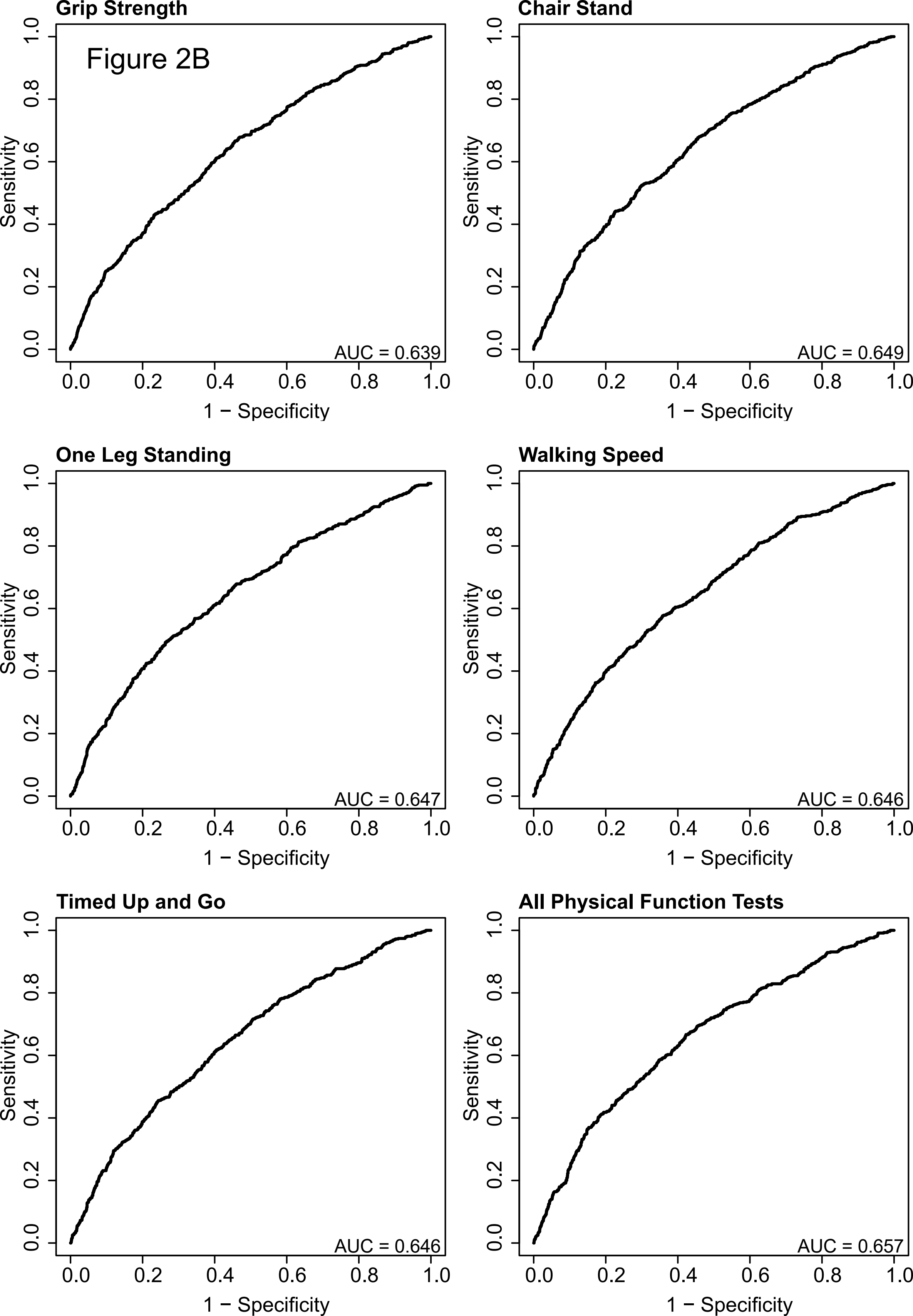
Figure 2B
Grip Strength
AUC = 0.639
Chair Stand
AUC = 0.649
One Leg Standing
AUC = 0.647
Walking Speed
AUC = 0.646
Timed Up and Go
AUC = 0.646
All Physical Function Tests
AUC = 0.657
Sensitivity
1 − Specificity
0.0
0.2
0.4
0.6
0.8
1.0

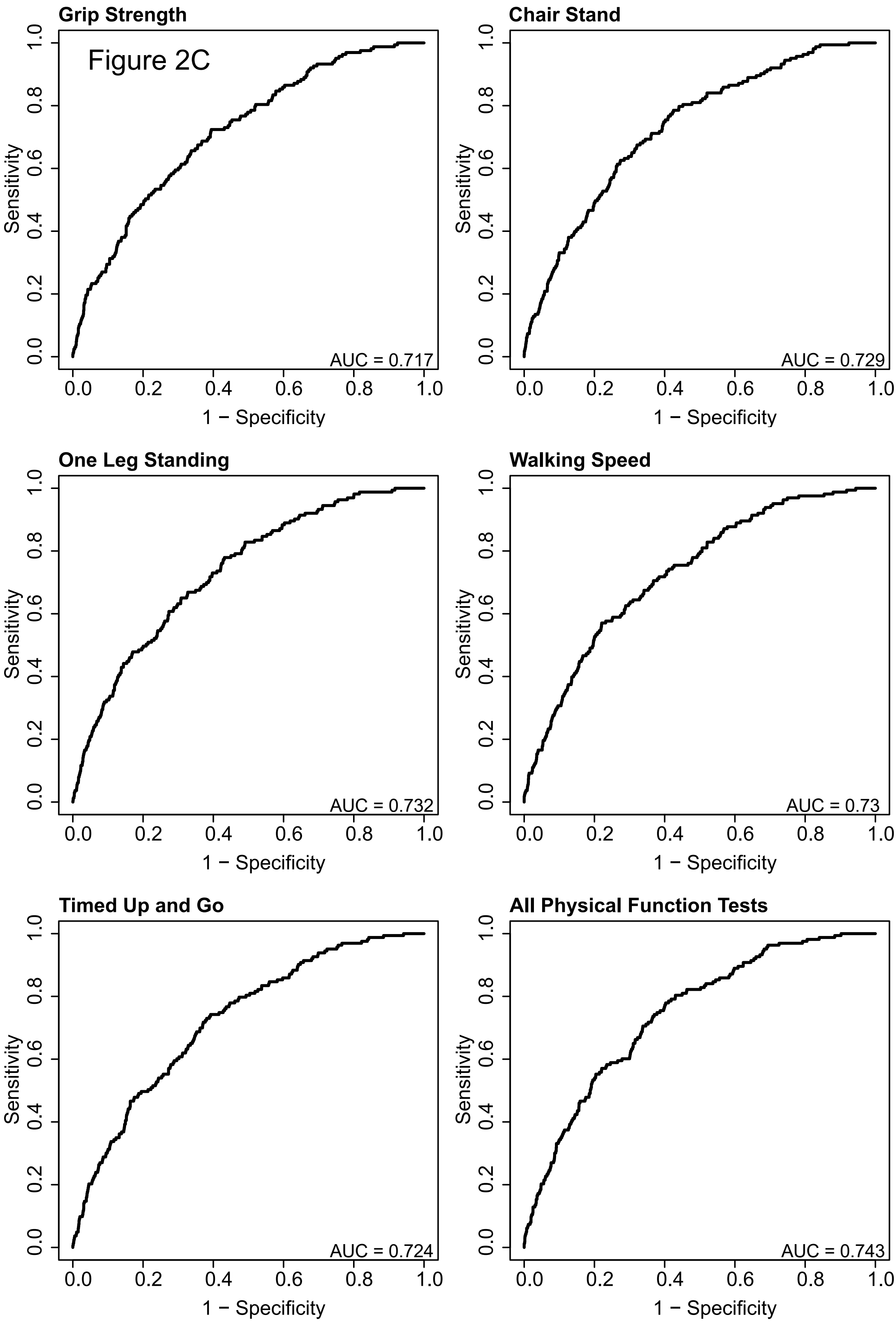

Figure 2C
Grip Strength
AUC = 0.717
Chair Stand
AUC = 0.729
One Leg Standing
AUC = 0.732
Walking Speed
AUC = 0.73
Timed Up and Go
AUC = 0.724
All Physical Function Tests
AUC = 0.743
Sensitivity
1 − Specificity
0.0
0.2
0.4
0.6
0.8
1.0
